# Autologous astigmatic corneal lenticule rotation with stromal excimer ablation for high hyperopic astigmatism: 2-year follow-up

**DOI:** 10.1177/11206721261444825

**Published:** 2026-04-17

**Authors:** Walter Sekundo, Suphi Taneri, Xingtao Zhou, Anke Messerschmidt-Roth

**Affiliations:** 1 Department of Ophthalmology, 61061 University Hospital Marburg (UKGM), Philipps University of Marburg, Marburg, Germany; 2 Eye Department at St. Francis Hospital, 74889 Center for Refractive Surgery, Muenster, Germany; 3 Ruhr-University Bochum, Bochum, Germany; 4 Eye Institute and Department of Ophthalmology, 159395 Eye & ENT Hospital, Fudan University, Shanghai, China

**Keywords:** Lenticule rotation, excimer ablation, shaping, combined surgery

## Abstract

**Purpose:**

To evaluate the long-term outcomes of a simultaneous lenticule rotation and excimer laser ablation for high hyperopic astigmatism, a technique addressing otherwise untreatable refractive errors.

**Methods:**

A 41-year-old female patient with a high refractive error (+3.75/−5.50 D) in her left eye underwent femtosecond laser-assisted FLEx, lenticule rotation, and excimer laser ablation. The procedure was performed to correct high astigmatism, which could not be treated with standard laser correction or phakic IOL implantation. Treatment parameters were adjusted to compensate for myopic shift due to centripetal lenticule shrinkage. Postoperative evaluations occurred at 3 months, 1 year, and 2 years.

**Results:**

Refractive astigmatism was reduced by >5.0 D, with corneal astigmatism decreasing by 3.7 D. The lenticule integrated well without distortions. Minor epithelial remodeling occurred, but no structural complications were observed. The patient achieved stable visual acuity and high satisfaction over 2 years.

**Conclusion:**

This technique provides a stable, reversible alternative for high hyperopic astigmatism, preserving accommodation and avoiding refractive lens exchange. Corneal lenticule surgery may offer a viable option for patients with extreme hyperopic astigmatism. Further clinical studies are warranted to confirm its long-term efficacy**.**

## Introduction

Since Sekundo et al. published their first report on Femtosecond Lenticule Extraction (FLEx), the byproduct of the surgery, a living tissue lens, the so-called corneal “lenticule” has been used for a variety of purposes.^1–3^ Despite a tremendous development of the Keratorefractive Lenticule Extraction (KLEx) in the last 2 decades with the recent approval of hyperopia and hyperopic astigmatism treatment the treatment range is limited.^4^ In practical terms, any astigmatism over 5 D cannot be fully corrected, especially if combined with hyperopia greater than +4 D. In 2019 Damgaard et al published their first experimental study on cadaver corneas with a genius idea of lenticule rotation.^5^ They cut a toric lenticule in the corneal stroma with a half of the desired astigmatic correction and rotated it by 90° achieving a full astigmatic correction. Shortly thereafter, Stodulka and Hjortdal proved this concept in a clinical setting.^6^ However, a lenticule rotation within a pocket is surgically challenging and lenticule stretching limited. Moreover, the thickness of the lenticule has to be increased significantly in order to allow grasping of the periphery without tearing the lenticule. Therefore, the authors of this communication prefer the FLEx over the KLEx technique for the correction of high hyperopic astigmatism. Having a flap and working in an open sky fashion facilitates a precise alignment of the lenticule along with a good bimanual stretching. The possibility to intrastromally ablate the cornea simultaneously with the rotation also opens a chance for one-step full range treatment as recently published by Shang et al.^7^ This approach might be more patient friendly then to perform a PRK on the cap after the healing of rotated lenticule as published by Stodulka et al.^8^ In all afore mentioned papers the excimer treatment was confined to myopias and high myopic astigmatism with a follow up after ablation not exceeding 6 months. Consequently, Shang wrote in her paper “this method is feasible and safe, with predictability requiring further study”. In this communication we close those 2 gaps: we show a 2-year outcome of a similar treatment, but in high astigmatism and hyperopia, and explain the calculations and the rational of the surgical technique used.

## Case report

In 2022, a 41-year-old female patient approached our clinic with the request for laser treatment for her high refractive error. Pre-operative tomography by Pentacam HR® and the tissue biomechanical index (TBI) of Corvis® (both Oculus GmbH/Germany) were within normal limits, with a corneal thickness of 533 μm in the right and 511 μm in the left eye. The corrected distance visual acuity (CDVA) OD was 1.0 decimal with the manifest refraction of −5.00/−1.00/9° and OS 0.8 decimal with +3.75/−5.50/1°. Due to the high refractive error the left eye had a mild degree of amblyopia. The patient was intolerant to rigid contact lenses.

While the right eye was eligible for a routine myopic KLEx (SMILE), the left eye did not qualify for any standard treatment: the anterior chamber depth was not sufficient for a hyperopic phakic intraocular lens, and the magnitude of astigmatism was too high for any conventional laser treatment. Hence, we offered a simultaneous lenticule rotation and excimer laser ablation as an off-label procedure for the OS. Table 1 displays the preoperative and the postoperative refraction at 3 months, 1 and 2 years.

**Table 1.** UDVA, refraction, and CDVA of the eye treated by autologous lenticule rotation and excimer ablation at each time interval.

	Autologous lenticule rotation and excimer ablation			
	Refraction	UDVA	OS	CDVA
pre-op	manifest		+3.75/−5.50/1°	0.8
	Cycloplegic (tropicamide)		+4.25/−5.50/179°	0.8
3 mo post-op	manifest	0.63	+0.25/−0.25/120°	0.63++
1-year post-op	manifest	0.63	+0.25/−1.0/140°	0.63
2-year post-op	manifest	0.63	+0.25/−0.25/140°	0.8

## Calculation of treatment parameters and surgical technique

Due to the patient's pre-presbyopic age and aiming rather for little overcorrection of hyperopia then undercorrection we chose to treat the tropicamide cycloplegic refraction of +4.25/−5.5cyl @ 1° (instead of 179°, the axis of the manifest refraction was chosen). The spherical equivalent (SEQ) of this refraction is +1.5 D. Our experience from the previous cases of pure lenticule rotation, i.e. without excimer reshaping, showed a concurrent myopic shift of +0.75 to +1.0 D.^9^ This phenomenon is believed to derive from the centripetal collagen shrinkage of the detached lenticule. Assuming the lenticule shrinkage would correct +0.75 D of hyperopia we decided to additionally ablate +1.0 D using excimer laser in order to achieve a postoperative refraction of −0.25 D.

The VisuMax 500 femtosecond laser (Carl Zeiss Meditec AG/Germany) was programmed for the standard SMILE treatment in the right eye using our proprietary nomogram and a 6.5 mm zone, 7.5 mm cap and 2.5 mm entering incision.

**Figure 1.**
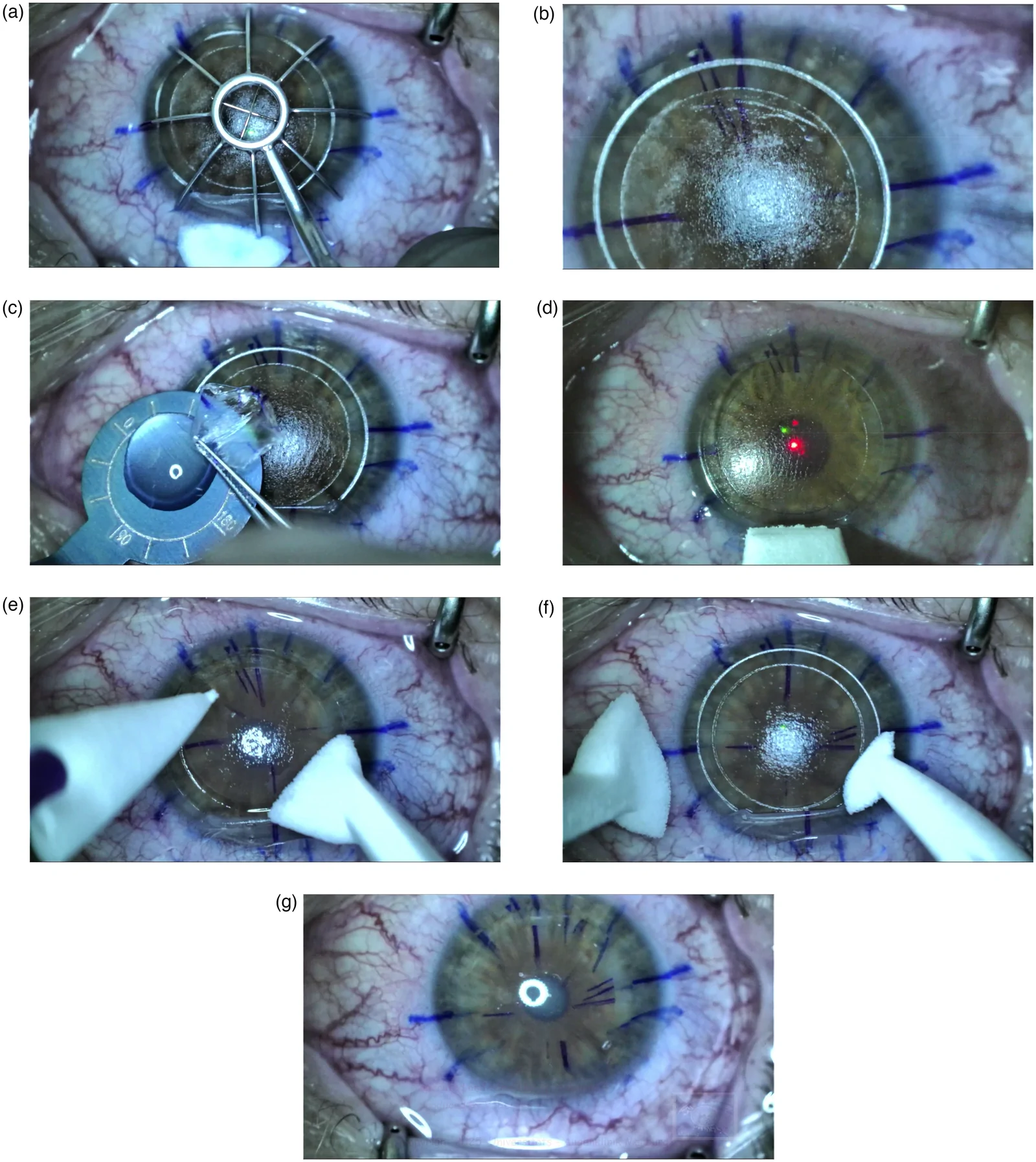
(a) A previously marked flap is reflected onto a wet sponge and the lenticule is marked with 4 spokes. (b) Two additional oblique marks at 5:45 o’clock position. (c) The lenticule is placed on the Zhou's lenticule spoon and (d) a hyperopic excimer ablation is performed. (e) The lenticule is placed back according to the marks and (f) rotated clockwise. (g) The flap is briskly flipped back using Zhou's waterbath technique.

For the left eye, the eye of interest in this report, the SMILE software was programmed as FLEx (with an 8.7 mm flap) and a 7.1 mm lenticule diameter including the 0.1 mm transition zone. The minimal thickness was purposely increased to 30 µm in order to have a stronger lenticule's edge. With these parameters the central lenticule thickness measured 90 µm. A M-size treatment pack was used. After the femtosecond cut was performed, the patient's bed was rotated under the MEL 90 excimer laser (Carl Zeiss Meditec AG/Germany). The eye tracker was locked in on the vertex. The surgeon (WS) marked and lifted the flap. In the next step, the lenticule was marked at 4 locations with two additional excentric lines at 5:45–5:50 o’clock position, dissected and placed aside onto Zhou's toric lenticule spoon. Stromal bed ablation of +1.0 D at a 7 mm optical zone was carried out removing 21 µm of tissue. The lenticule was placed onto the stromal bed according to the initial marks and then rotated 90°clockwise and stretched bimanually with two spear sponges in all directions to fit snugly onto the imprint on the stromal bed. Once the lenticule was immobile, the flap was repositioned using Zhou's “water bath one-flip-technique” (Figure 1a–g). The flap edges were adjusted to the radial marks and a bandage contact lens soaked in a preservative free antibiotic and steroid was placed on the eye's surface.

The postoperative course was uneventful in both eyes, however, expectedly the left eye took at least one week longer for the cornea to fully recover.

**Figure 2.**
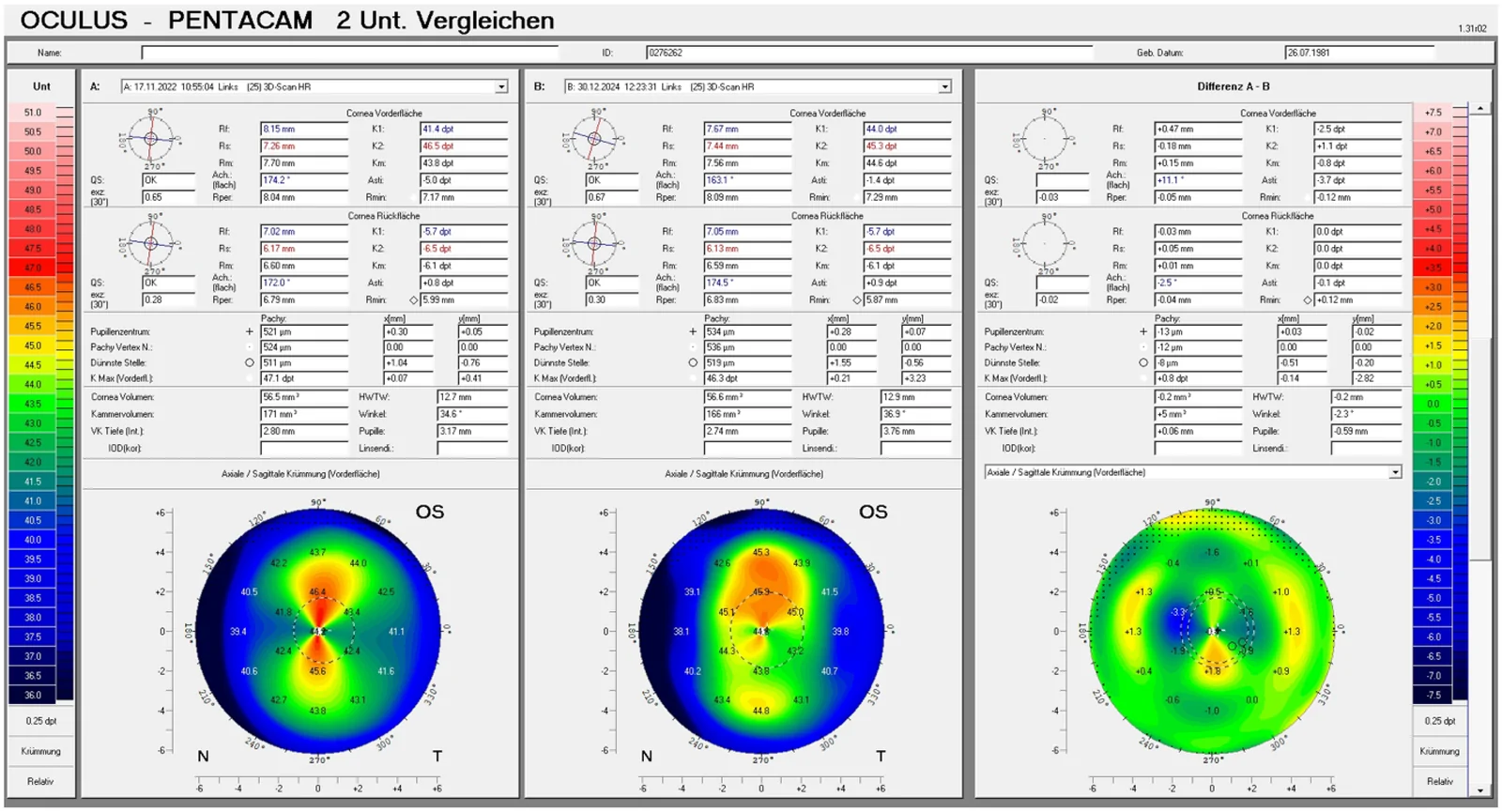
A differential map (Pentacam HR, Oculus GmbH, Germany) of the anterior surface (sagittal map) of the cornea prior to surgery and 2 years later. A reduction of 3.7 D cyl can be appreciated.

**Figure 3.**
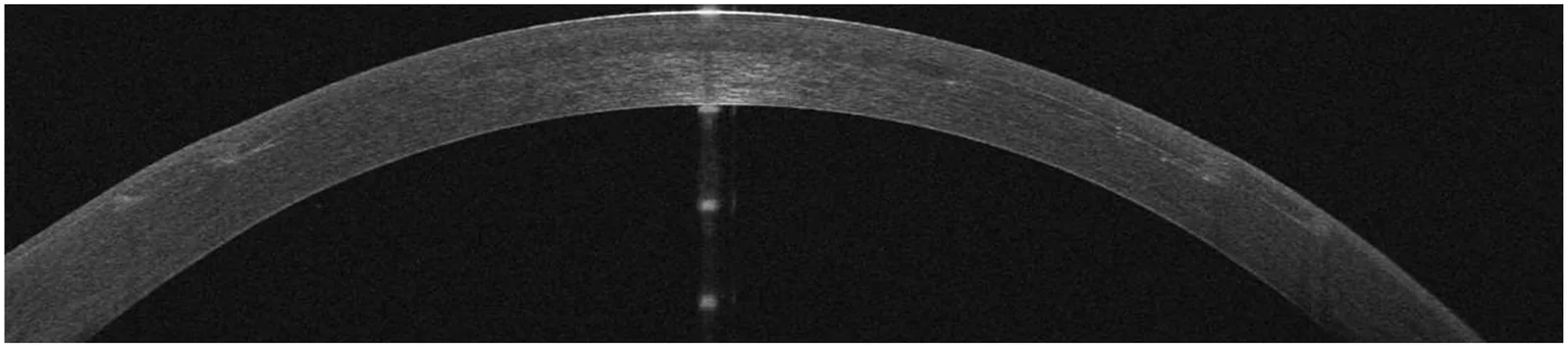
An OCT line (Avanti Optovue, USA) shows a smooth integration of the lenticule and faint scars at the flap's edge.

**Figure 4.**
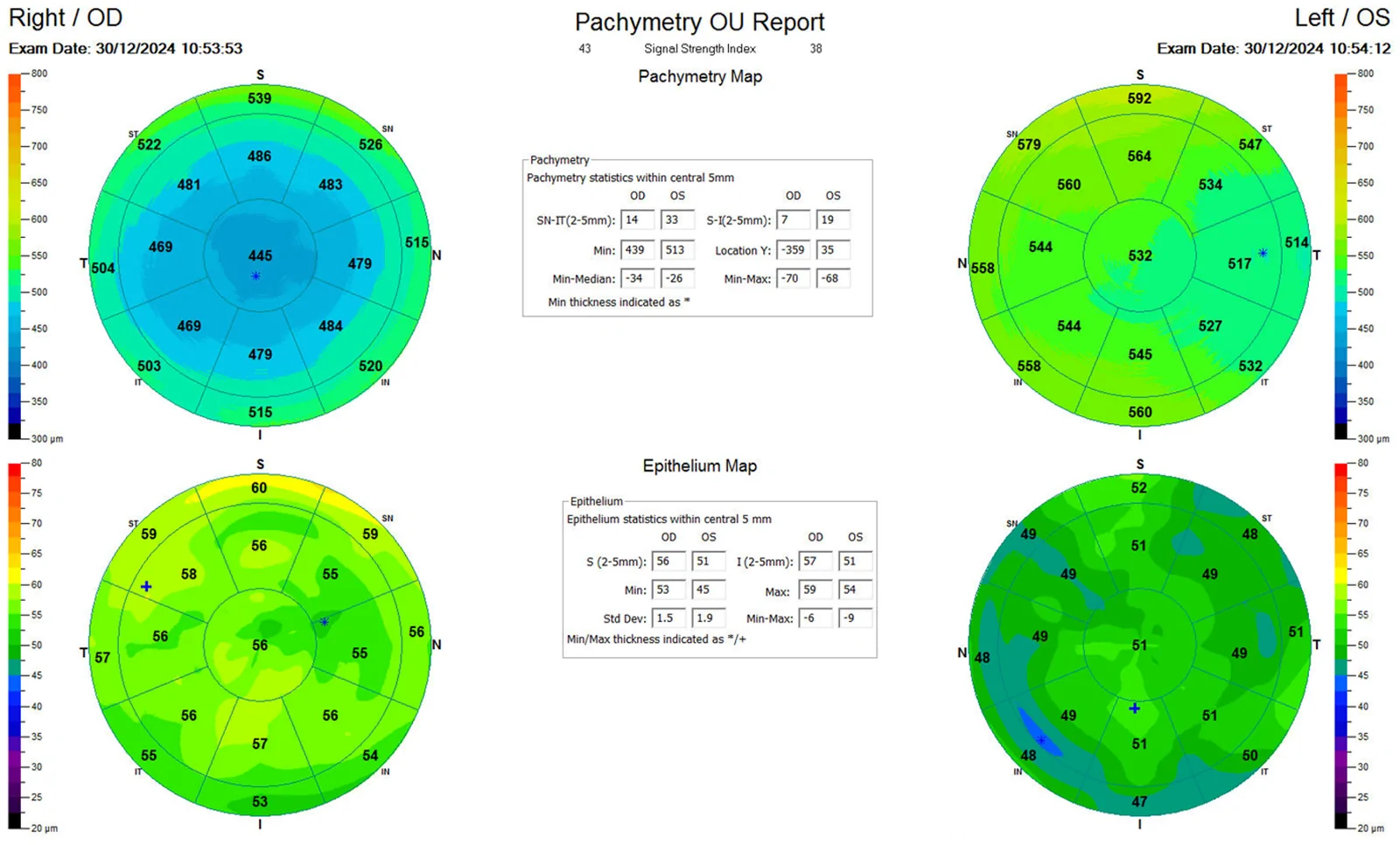
OCT epithelial map (Avanti Optovue, USA) shows a different epithelial response to the procedures: While the right eye shows central epithelial thickening after a myopic SMILE the left eye demonstrates a modest peripheral epithelial thinning to be expected after a hyperopic correction.

Table 1 shows a remarkable reduction of refractive astigmatism of over 5 D cyl. Also, the corneal astigmatism was reduced by 3.7 D cyl (Figure 2). The lenticule was perfectly embedded with corneal stroma without any wrinkles or distortions. Tiny scars were present at one location at the flap's edge (Figure 3). There was a modest epithelial remodeling with some compensational decrease at the periphery (Figure 4). The patient was delighted with both the uncorrected and best corrected vision.

## Discussion

As recently shown by Shang et al. at combined lenticule reshaping and rotation offers an excellent method to correct otherwise untreatable refractive errors. However, in their study of 6 eyes all eyes treated were myopic. Because of this it made very much sense to ablate the center of the respective lenticule first and then rotate. This maneuver saves tissue and preserves a uniform stromal hydration for excimer ablation. The combination of hyperopia and astigmatism, as in the present case, poses an entirely different challenge. Hyperopic ablation of +1.0 D using 7 mm zone removes 21 µm of tissue in the mid periphery of the cornea radiating up to 9 mm as a transition zone. The risk is that this ablation would reduce the lenticule's edge thickness down to 10 µm endangering the integrity of the lenticule during the subsequent dissection from the stromal bed. This was the reason to remove the lenticule first, perform the ablation and replace it 90° rotated.

The study by Shang et al. had a follow-up period of 6 months. The present case highlights a remarkable refractive stability over 2 years. A slight deterioration of the CDVA and the refraction at 1 year follow-up can be attributed to the secondary ocular surface dryness aggravated by the cold weather at the time of examination.

Our case is a good example that a simultaneous autologous astigmatic lenticule rotation and excimer ablation using the above-mentioned calculation and technique leads to a predictable and stable refraction over the period of 2 years not only in myopia, but also in hyperopia combined with high astigmatism. Moreover, unlike in myopia, where a combination of a phakic IOL and a subsequent laser correction of the remaining astigmatism is a viable option, hyperopic eyes do typically not have an adequate anterior chamber depth for the implantation of a phakic lens. This leaves these eyes with the standard option of refractive lens exchange only, which in our opinion is unethical in a younger population due to the loss of accommodative capacity. In these cases, corneal lenticular surgery offers the only (at least partially) reversible and stable solution. A touch-up using surface ablation is another possibility to enhance the outcome if needed.^8^
